# The efficacy and molecular mechanism of the effect of schisandrin b on the treatment of erectile dysfunction 

**DOI:** 10.22038/ijbms.2019.27455.6701

**Published:** 2019-08

**Authors:** Wei Liu, Chen Zhao, Yanping Huang, Yidong Liu, Mujun Lu

**Affiliations:** 1 Department of Urology and Andrology, Renji Hospital, Shanghai Jiao Tong University, School of Medicine, Shanghai 200001, People’s Republic of China

**Keywords:** cAMP-PKA pathway, Cyclic nucleotides, Erectile dysfunction Intracavernosum pressure NO-cGMP pathway, Schisandrin b

## Abstract

**Objective(s):**

The purpose of this study is to determine the efficacy and molecular mechanism of the effect of schisandrin b (SCHB) on treating erectile dysfunction (ED) in a rat model with bilateral cavernous crushing nerve injury.

**Materials and Methods:**

The ED rat model was established with bilateral cavernous nerve crushing, and then confirmed by apomorphine. Fifty healthy eight-week-old ED rats were randomly assigned into five group, including control group (sham surgery), bilateral cavernous nerve crushing injury group (BCNC), BCNC with low SCHB (100 mg/d), BCNC with medium SCHB (200 mg/d) and BCNC with high SCHB (400 mg/d). For the last three groups, SCHB was given for 2 months. Then, we examined intracavernosal pressure (ICP), cyclic nucleotides (cAMP, cGMP), endothelial nitric oxide synthase (eNOS) and neuronal NOS (nNOS) in all groups.

**Results:**

In the study of ICP, SCHB was able to improve ED in a dose-dependent manner. In addition, as compared to the BCNC group, the relative expression of eNOS and nNOS in medium and high concentration of SCHB-treated groups are higher than BCNC group. Moreover, all groups treated with SCHB showed a significant higher expression level of cAMP and cGMP.

**Conclusion:**

These results suggested that SCHB were able to significantly improve the ED on rat model through the NO-cGMP and cAMP- protein kinase A (PKA) pathway.

## Introduction

A subtle change between the pro-contraction and anti-contraction factors critically reflects on the penis erection. Improvement of erection can be achieved by enhancing the cavernous muscle relaxation or inhibiting contraction. It has already been identified that nitric oxide (NO)-cyclic guanosine monophosphate (NO-cGMP) pathway plays a critical role in the process of erection (1). After sex stimulation from brain or penis itself, the messages are sent to the cavernous muscle through parasympathetic nerve, which can release acetylcholine. Then, endothelial nitric oxide synthase (eNOS) in the endothelial cell is activated and converts L-arginine to neuronal NOS (nNOS), which can also be accomplished by nNOS produced by nonadrenergic-noncholinergic (NANC) neurons. NO enters into the smooth muscle cells to activate guanylyl cyclase turning guanosine triphosphate (GTP) to cGMP, which can activate protein kinase G (PKG). PKG closes calcium channel while opens potassium channel in order to lower calcium concentration in cytoplasm. As a result, the smooth muscle relaxes and penis erects (2).

Cyclic adenosine monophosphate (cAMP), an important second messenger, activates protein kinase A (PKA) leading to potassium channel opening and calcium channel closing, which can decrease the concentration of calcium in the cytoplasm. With the decrease of calcium, calcium dependent myosin detached from actin, which results in relaxation of cavernous muscle (3).

In the 90^th^ decade, the invention of phosphodiesterase type 5 (PDE 5) inhibitors began a new approach in treatment for erectile dysfunction (ED), which made it the first line drug ever since (4). However, PDE 5 inhibitors have its own side effects, including headache, dizziness, and visual and hearing changes. Also, there are about 11% to 44% patients who are not sensitive to PDE 5 inhibitors (5). So, it becomes more important to combine PDE 5 inhibitors and second line treatment like vacuum erection device (VED), ICI, and adrenergic receptor inhibitors etc.

In America, prostate cancer is the most common cancer and the second cause of cancer death in male (6). Retropubic radical prostatectomy is the standard approach for most patients who choose surgery for the definitive management of their prostate cancer and the prognosis is good (7, 8). The most important frequent complications associated with radical retropubic prostatectomy include urinary incontinence and ED (9, 10). With the development of minimally invasive (robotic or laparoscopic) technique, the incidence of urinary incontinence gradually decreases to an acceptable level (11, 12). However, the ED is still a black cloud hanging over those patients head. According to American Urological Association (AUA), men after radical prostatectomy have an ED prevalence of 10% to 100% (13). As the young prostate cancer patients increase, the rehabilitation therapy for patients after radical prostatectomy is urgently required (7).

Recently, many studies have proved that *schisandrin b *(SCHB) has anti-inflammatory, anti-oxidant and anti-cancer functions (14-16). But, its effects on ED are not further studied. Previous researches show that the extract of *Schisandra chinesis* has a significant relaxant effect on isolated cavernosum smooth muscle of rabbit *in vitro. *And SCHB is then found to be the major lignan constituents, which also shows synergistic phenomenon with sildenafil (17). So based on the *in vitro* studies, we want to explore the SCHB’s efficacy on experimental animals and further elucidate the mechanism in molecular level.

## Materials and Methods

***Rats ED model preparation and grouping***

Two-month-old male Sprague Dawley rats with weight approximately 250 g were proved to have normal sexual function after sexual intercourse. Then, these rats were divided into five groups: the control group (n=10), the bilateral cavernous nerve crushing group (BCNC group, n=10), the administration of SCHB (100 mg/d) following BCNC (BCNC+low SCHB, n=10), the administration of SCHB (200 mg/d) following BCNC (BCNC+medium SCHB, n=10), the administration of SCHB (400 mg/d) following BCNC (BCNC+high *SCHB*, n=10). One day after surgery, all rats would be injected subcutaneously with apomorphine (APO, 100 ug/kg), (Sigma), with concentration of 40 ug/ml. In the following 30 minutes, the rats would be eliminated from the study if there were any signs of erection in the termination of penises.

***Drug dosage and time interval***

Two days after surgery, low SCHB (100 mg/d), medium SCHB (200 mg/d), and high SCHB (400 mg/d) were orally administrated, respectively. At the same time, control and BCNC groups were given the exact same food except for SCHB. All five groups raised for 2 months.

***Measurement of intracavernosal pressure***

The rats were anesthetized, and then a midline abdominal incision was made to access MPG posterolateral to prostate gland. The penis was dissected and the corpus cavernosum and crus of the penis were exposed fully. In order to get intracavernosal pressure (ICP), a heparinized 25G butterfly needle connected to Powerlab/4SP was inserted in the corpus cavernosum of penile. Then, an electrical stimulator was place on the cavernous nerve at 15 HZ, 1.2 ms, with the voltage at 2.5 V, 5 V and 7.5 V for 1 min, respectively. The interval between stimulations was about 10 min. Following the complete functional analysis, the penises were excised and washed twice in phosphate-buffered saline (PBS), the middle portion of penis were kept in CryoTubes, and the remaining bodies were frozen at -80 ^°^C in the refrigerator.

**Figure 1:**
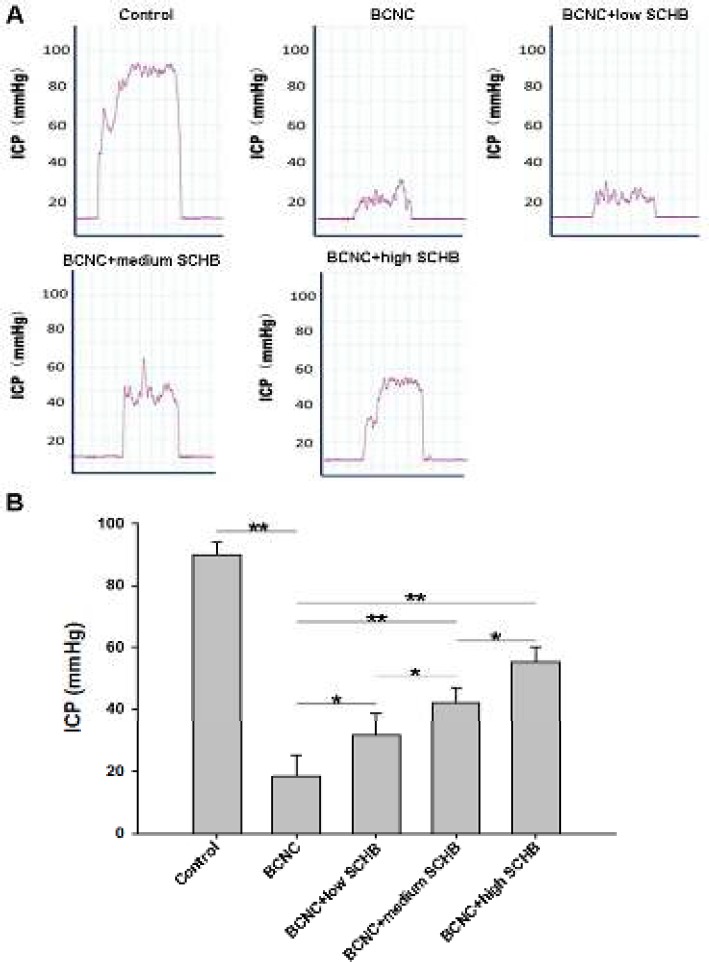
The intracavernosal pressure (ICP) evaluation of five groups respectively. (A) The representative ICP curve of each group (n=10). (B) The statistical analysis of ICP of each group. *P<0.05, **P<0.01

**Figure 2:**
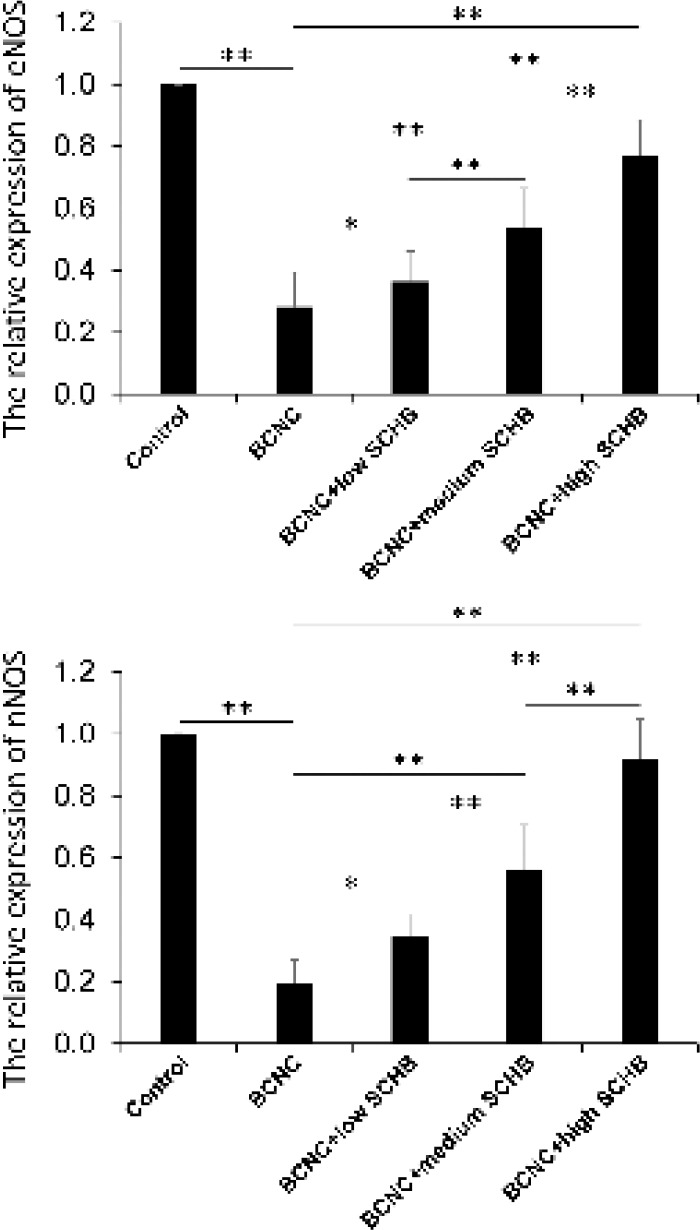
The relative expression of endothelial nitric oxide synthase (eNOS) and neuronal NOS (nNOS) among the five groups detecting by RT-PCR. *P<0.05, **P<0.01

**Figure 3:**
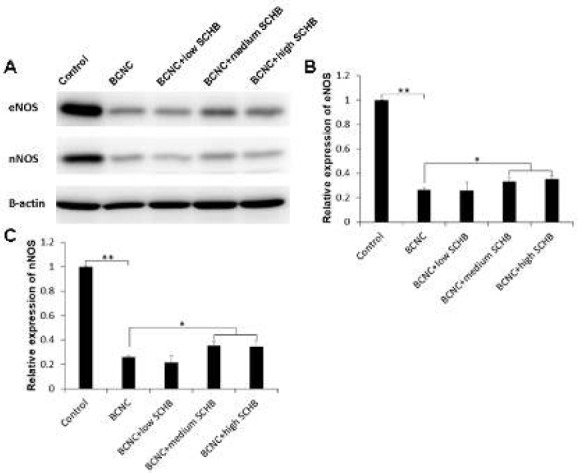
(A) The relative expression of endothelial nitric oxide synthase (eNOS) and neuronal NOS (nNOS) among the five groups detecting by Western blot method. The quantification of eNOS (B) and nNOS (C) expression among the five groups. *P<0.05, **P<0.01

**Figure 4:**
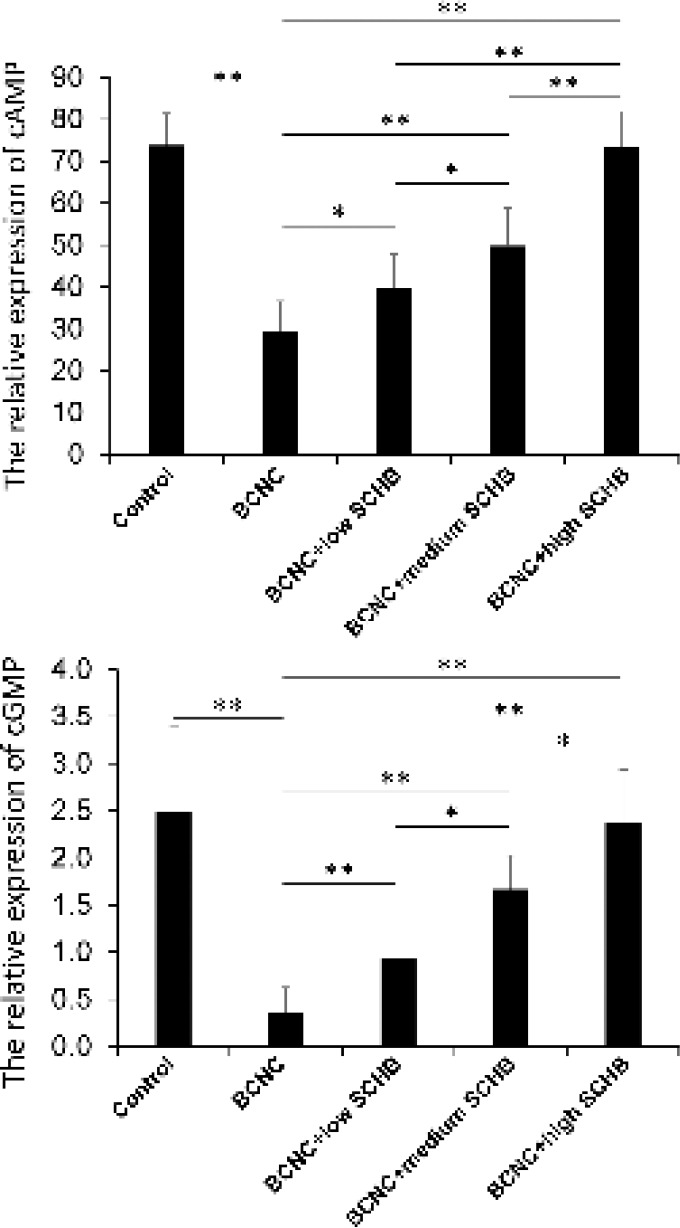
The relative expression of cyclic adenosine monophosphate (cAMP) and cyclic guanosine monophosphate (cGMP) among the five groups detecting by radioimmunoassay. *P<0.05, **P<0.01

***RT-PCR***

Total RNA was extracted from rat PCC using TRIZOL reagent (Invitrogen, Carlsbad, CA) and reverse transcription was performed using Superscript II and 18-mers Oligo-dT (Takara, Japan). Specific primers were designed using primer express software (Applied Biosystems, Carlsbad, CA) and their primer sequences were as follows: rat eNOS, 5’-TCGTCCCTGTGGAAAGACAAG-3’ (forward) and 5’-CTTTGGCGAGCTGGTAACTGT-3’ (reverse); rat nNOS, 5’-GAGAAGCAACGCCTGTTGGT-3’ (forward) and 5’-CCCCATTTCCACTCCTCGTA-3’ (reverse); and rat actin, 5’-TGGCATCCTGACGCTCAA-3’ (forward) and 5’-TCGTCCCAGTTGGTCACGAT-3’ (reverse). The real-time PCR reaction performed in a final volume of 10 µl, containing 10 ng of reverse transcribed total RNA, 200 nM of forward and reverse primers and 2×PCR master mix. PCR reaction was carried out in 384-well plates using the ABI Prism 7900HT Sequence detection System (Applied Biosystems). All reactions were performed in triplicate.

***Western blot***

The removed PCC tissue was homogenized in ice-cold buffer containing 0.32 M sucrose, 0.2 M HEPES (pH 7.4), 1 mM ethylenediaminetetraacetic acid (EDTA), 1 mM dithiothreitol (DTT), 10 μg/ml leupeptin, 2 μg/ml aprotinin, 1 μg/ml pepstatin, 10 μg/ml trypsin inhibitor and 1 mM phenylmethylsulfonyl fluoride (PMSF). The homogenized solution was placed on ice for 15 min, and then centrifuged at 4 ^°^C for 13000 rpm for another 30 min, and the supernatant was separated. Bovine serum albumin was used as separating solution. Thirty μg of the quantitative protein was denatured at 95^ °^C for 5 min and electrophoresis was performed on a 6% discontinuous sodium dodecylsulfate-polyacrylamide gel (SDS-PAGE). The proteins were then electroblotted onto a 0.2 μM polyvinylidenedifluoride (PVDF) membrane (Amersham Biosciences, Piscataway, NJ, USA) for 150 min at 25 V. The membranes were reacted with blocking buffer (5% skim milk in TBS-T buffer) for 30 min at ambient temperature. eNOS antibody (Genscript, Piscataway, NJ, USA) and nNOS antibody (Abcam, Cambridge, MA, USA) were reacted for 12 hr at 4 ^°^C, and the membrane was washed three times using Tris-buffered saline with Tween (TBST) at intervals of 10 min. As the secondary antibodies, anti-mouse IgG-HRP and anti-goat IgG-HRP (1:2000 dilution; Zymed Laboratories, San Francisco, CA, USA) were reacted at ambient temperature for 1 hr, and the membrane was washed again with TBST six times with an interval of 5 min between each washing. Chemiluminescence was detected using ECL western blotting detection reagents.

***Radioimmunoassay (RIA) of cGMP and cAMP concentration***

For measurement of the cGMP and cAMP concentration in PCC, the samples were minced in 2 ml of ice-cold trichloroacetic acid and homogenized at 4 ^°^C with three 30 sec bursts in a Polytron homogenizer. The homogenates were centrifuged at 1000 g for 10 min at 4 ^°^C, and the supernatant was extracted with ether and dried. The pellet was treated with 500 µl NaOH (1 N), ultrasonicated, and used for protein determination. Levels of cGMP and cAMP were measured with a specific RIA, as described previously (18). Briefly, standards or samples were taken up in a final volume of 100 µl of 50 mM sodium acetate buffer (pH 4.8) containing theophylline (8 mM), then 100 µl of diluted cGMP antiserum (Calbiochem-Novabiochem) and iodinated 2’-O-monosuccinyl-guanosine 3’,5’-cyclic monophosphate tyrosyl methyl ester (125 I-ScGMP-TME; 10,000 counts/min [cpm] per 100 µl) were added for the measurement of cGMP and incubated for 24 hr at 4 ^°^C. For the acetylation reaction, 5 µl of a mixture of acetic anhydride and triethylamine (1:2 dilution) were added to the assay tube before antiserum and tracer were also added. The bound form was separated from the free form by charcoal suspension. The amount of cGMP was expressed as femtomol per milligram of PCC tissue.

***Statistical analysis***

The results are expressed as the mean ± standard deviation (SD). The statistical significance of differences was calculated by one-way analysis of variance (ANOVA), followed by Bonferroni’s multiple comparison test. Concentration-dependent responses before and after the treatment with blockers were compared by Student’s paired t-test. A *P<*0.05 was considered significant.

## Results

***Evaluation of ICP***

As shown in Figure 1, ICP response to the stimulation of the cavernous nerve was much higher in control group (89.866±4.0929 mmHg) compared to BCNC group (18.404±6.704 mmHg) (*P*<0.01). However, as compared to BCNC group, significantly increase in ICP response could be found in BCNC+low SCHB group (31.552±7.0536 mmHg) (*P*<0.05), BCNC+medium SCHB group (42.133±4.7885 mmHg) (*P*<0.01) and BCNC+ high SCHB group (55.295±4.6646 mmHg) (*P*<0.01). And the ICP response increased with the increased concentration of SCHB*. *These results suggested that SCHB was able to improve erectile function in a dose-dependent manner.

***RT-PCR***

The relative mRNA expression of nNOS and eNOS were shown in Figure 2. The expression of nNOS and eNOS in BCNC group was much lower than control group (*P*<0.01). At the same time, the expression of nNOS and eNOS in BCNC+low SCHB, BCNC+medium SCHB and BCNC + high *SCHB* groups were gradually increased with the increased concentration of SCHB when compared to BCNC group (*P*<0.01). Moreover, the expression of nNOS in BCNC+high SCHB group almost reached control level.

***Western blot***

In order to further determine the expression of eNOS and nNOS among the five groups, we detected the expression of eNOS and nNOS at protein level by Western Blot method. As shown in Figure 3, it was similar to mRNA expression results.

The expression of eNOS and nNOS in BCNC group were significant lower than that in control group. In addition, the expression of eNOS and nNOS in BCNC +medium SCHB, and BCNC+high SCHB groups were higher than that in BCNC group. 

***Radioimmunoassay (RIA) of cGMP and cAMP concentration***

As shown in Figure 4, from the control group to BCNC +high SCHB group, the concentration of cAMP is 73.782 ±7.6185, 29.349±7.2653, 39.579±8.2215, 49.776± 8.9768, and 73.322±8.5251 pmol/mg, respectively, and the concentration of cGMP is 2.497±0.8914, 0.358 ±0.2658, 0.937±0.4859, 1.669±0.3573, and 2.384± 0.5533 pmol/mg, respectively. The expression of cAMP and cGMP in control group was higher than BCNC group (*P<*0.01). Also, the recovery of cAMP and cGMP expression in BCNC+low SCHB, BCNC+medium SCHB and BCNC+high SCHB groups were obviously higher than BCNC group (*P<*0.01). In addition, expression of both cAMP and cGMP increased in a dose dependent manner. 

## Discussion

***The efficacy of SCHB on ED in rat model***

Erection of the penis is a complicated process instead of a simple infusion of blood. From the relaxation of cavernous smooth muscle and reduced venous outflow to acquire and maintain a firm erection, it involves the velocity of blood flow, ICP, and changes of cavernous smooth muscle and intracavernosal plexus. Essentially, erection of penis is complicated venous activity under the control of neuroendocrine regulation, which can only be acquired by a subtle cooperation of neuroendocrine system, cavernous muscle and other psychological factors.

ICP is the gold standard for evaluation of erection, so in this study, we used ICP to evaluate the function recovery. We found that 2 months after the surgery, erection was not recovered in BCNC group when compared to sham surgery group (*P*<0.01). But, with the help of SCHB (BCNC+low SCHB, BCNC+medium SCHB and BCNC+ high SCHB groups), a significance recovery in ICP was observed compared to BCNC group (P<0.01). And the recovery level of ICP indicated a positive correlation with the concentration of SCHB. It can be further inferred that with administration of SCHB for 2 months, ED of rats with nerve injury can be improved in a dose dependent manner. However, even with administration of high dose of SCHB (400 mg/d) (BCNC+high SCHB group), there is still a significant difference in ICP between control and BCNC+high SCHB group (*P*<0.01), suggesting that SCHB could improve the recovery of ED in on rat model with bilateral cavernous nerve crushing injury, but it cannot recover erectile function to original level.

***The effect of SCHB on NO-cGMP signal pathway activation in rat ED model***

The contraction and relaxation of cavernous smooth muscle is under the control of adrenergic, cholinergic, and non-adrenergic-non-cholinergic (NANC) neurotransmitters, which play the major role here. Nitrogen monoxide (NO) is the most important NANC neurotransmitter and plays an important role in the L-arginine- NO- cGMP pathway. As a gas messenger, NO can be transformed to L-arginine via NOS. The lipid soluble NO combines to Fe^2+^ in guanylyl cyclase, which leads to conformation change of the enzyme. The changed cyclase catalyzes the production of cGMP from GTP. PKG is then activated to inhibit influx of calcium by closing calcium channel. As a result, NO helps the cavernous smooth muscle to relax (19).

The half-life period of NO is quite short, which makes it hard to study. NOS, the rate-limiting enzyme, is used to study the NO- cGMP pathway. NOS can be divided into two groups including nNOS and eNOS. Neuronal NOS is primarily responsible for the generation of NO in the nerve fibers, while eNOS primarily acts in the vascular endothelial cells. In the penile tissue, the expression of eNOS is higher than nNOS (20). Studies indicated that nNOS knock-out rats could acquire erection with compensated increase in eNOS (21). NOS also plays role in the pathological processes; it is found that NOS decreases in rats with diabetic ED and neural injury ED (21, 22).

In the present study, as compared to BCNC group, the control group had a high mRNA and protein expression of nNOS, eNOS, and cGMP (*P*<0.01), while the groups with SCHB (BCNC+low SCHB, BCNC+medium SCHB and BCNC+high SCHB) showed significant increase of mRNA and protein expression of nNOS, eNOS, and cGMP level, which indicated that SCHB helped the cavernous tissue to restore eNOS, nNOS and cGMP in some extent. We assumed that it might make a difference in the expression of eNOS and nNOS, which lead to increase of NO, cGMP and smooth muscle relaxation. In the three groups with administration of SCHB, the increase in NOS expression revealed a positive correlation with the increase of cGMP, which was strong evidence that SCHB improved ED via NOS-cGMP pathway. However, despite the NOS-cGMP pathway, further research should be conducted to understand the relation of SCHB and PDE, which might play an important role in the improvement of SCHB on ED.

***The effect of SCHB on NO-cAMP signal pathway in rat ED***

cAMP- PKA is a classic pathway in which the G-coupled protein (Gs and Gi) controls the level of cAMP via adenylate cyclase. With the changes of cAMP, PKA, which is responsible for the phosphorylation of many proteins, is activated or deactivated. And, the potassium and calcium channels are controlled by activated PKA, so the calcium level in the cytoplasm decreases, leading to detachment of myosin from actin, which means relaxation of smooth muscle (3).

In the present study, cAMP in the BCNC group was much lower than control group (*P*<0.01). However, with the help of SCHB, cAMP in BCNC+low SCHB, BCNC +medium SCHB and BCNC+high SCHB groups were significant increased compared to BCNC group (*P*<0.01). And, there was a positive correlation between the dose of SCHB and the level of cAMP.

The results showed that after BCNC injury, the level of cAMP decreases, but with the administration of SCHB, all rats with BCNC injury showed higher level of cAMP, which suggested that SCHB might increase cAMP via cAMP-PKA pathway to relax cavernous smooth muscle and to acquire erection.

## Conclusion

In this study, BCNC injury was made in rat model of ED, and SCHB was used to treat these rats. The ICP results suggested that SCHB was able to relieve ED in a dose-dependent manner. In addition, as compared to the BCNC group, the groups with SCHB treatment showed significant higher levels of cAMP and cGMP, as well as rise in both mRNA and protein expression of eNOS and nNOS. These results suggested that SCHB was able to significantly improve ED on rats with nerve injury through the NO-cGMP and cAMP-PKA pathways. However, this study has limitation; for example, this study lacks a positive control. In our further study, it will be added.

## Declaration of Interest

The authors state that there is no competing interest.
